# Erratum: Confirmation of the topology of the Wendelstein 7-X magnetic field to better than 1:100,000

**DOI:** 10.1038/ncomms14491

**Published:** 2017-02-14

**Authors:** T. Sunn Pedersen, M. Otte, S. Lazerson, P. Helander, S. Bozhenkov, C. Biedermann, T. Klinger, R. C. Wolf, H-S Bosch, The Wendelstein 7-X Team

Nature Communications 7: Article number: 13493; DOI: 10.1038/ncomms13493 (2016); Published: 11 30 2016; Updated: 02 14 2017

The original version of this Article contained errors in the spelling of authors names and their affiliations. These errors were:

The affiliation of Boyd Blackwell is 10, but was incorrectly given as 10,11,12.

In the list of the Wendelstein 7-X (W7-X) team members, the name of Matthias Borchardt was repeated twice; one instance has been removed.

In the list of the Wendelstein 7-X (W7-X) team members, the name `̀Shaocheng Liu'' was repeated twice and, in one instance, mistakenly spelled as `̀Shoacheng Lui'' The misspelled instance has been removed.

The name of Paul van Eeten was incorrectly spelled as Paul von Eeten.

The affiliation of Waldemar Figacz is 17, but was incorrectly given as 1.

The name of Stefan Heinrich was incorrectly spelled as Stefan Heirnich.

The affiliation of Winfried Kernbichler is 24, but was incorrectly given as 24,25.

The affiliation of Matt Landreman is 12, but was incorrectly given as 1.

The name of Henning Maaßerg was incorrectly spelled as Henning Maassberg.

The affiliation of Kieran McCarthy is 6, but was incorrectly given as 1.

The name of George Hutch Neilson was incorrectly spelled as G Hutch Neilson.

The affiliation of Jef Ongena is 36, but was incorrectly given as 9.

The affiliation of Hans Oosterbeek is 1, but was incorrectly given as 5.

The name of Maria Ester Puiatti was incorrectly spelled as Maria Ester Piulatti.

The name of Konrad Riße was incorrectly spelled as Konrad Risse.

The name of Joaquim Loizu Cisquella was incorrectly spelled as Joaquin Loizu Cisquella.

The name of Ryosuke Seki was incorrectly spelled as Ryoshuke Seki.

The affiliation of Matthew Stoneking is 25, but was incorrectly given as 1.

In the list of W7-X team members, the name of Adrian von Stechow was repeated twice; one instance has been removed.

The name of Christian Perez von Thun was incorrectly spelled as Christian Perez Von Thun.

The name of Tran-Thanh Ngo was mistakenly spelled as Tran-Tranh Ngo.

The affiliation of Adelle Wright is 10, but was incorrectly given as 10,11,12.

The affiliation of Glen Wurden is 11, but was incorrectly given as 1.

The affiliation of Masayuki Yokoyama is 23, but was incorrectly given as 1.

In affiliation 18, the Istituto di Fisica del Plasma “Piero Caldirola” was incorrectly spelled Istituto di Fisica del Plasma Piero Caldirola”.

These have now been corrected in both the PDF and HTML versions of the Article.

